# The Effect of High Ambient Temperature on the Elderly Population in Three Regions of Sweden

**DOI:** 10.3390/ijerph7062607

**Published:** 2010-06-14

**Authors:** Joacim Rocklöv, Bertil Forsberg

**Affiliations:** Department of Public Health and Clinical Medicine, Occupational and Environmental Medicine, Umeå University, SE-901 87 Umeå, Sweden; E-Mail: Bertil.Forsberg@envmed.umu.se

**Keywords:** mortality, temperature, heat, heat waves, weather, climate change, public health, death, humidity

## Abstract

The short-term effects of high temperatures are a serious concern in the context of climate change. In areas that today have mild climates the research activity has been rather limited, despite the fact that differences in temperature susceptibility will play a fundamental role in understanding the exposure, acclimatization, adaptation and health risks of a changing climate. In addition, many studies employ biometeorological indexes without careful investigation of the regional heterogeneity in the impact of relative humidity. We aimed to investigate the effects of summer temperature and relative humidity and regional differences in three regions of Sweden allowing for heterogeneity of the effect over the scale of summer temperature. To do so, we collected mortality data for ages 65+ from Stockholm, Göteborg and Skåne from the Swedish National Board of Health and Welfare and the Swedish Meteorological and Hydrological Institute for the years 1998 through 2005. In Stockholm and Skåne on average 22 deaths per day occurred, while in Göteborg the mean frequency of daily deaths was 10. We fitted time-series regression models to estimate relative risks of high ambient temperatures on daily mortality using smooth functions to control for confounders, and estimated non-linear effects of exposure while allowing for auto-regressive correlation of observations within summers. The effect of temperature on mortality was found distributed over the same or following day, with statistically significant cumulative combined relative risk of about 5.1% (CI = 0.3, 10.1) per °C above the 90th percentile of summer temperature. The effect of high relative humidity was statistically significant in only one of the regions, as was the effect of relative humidity (above 80th percentile) and temperature (above 90th percentile). In the southernmost region studied there appeared to be a significant increase in mortality with decreasing low summer temperatures that was not apparent in the two more northerly situated regions. The effects of warm temperatures on the elderly population in Sweden are rather strong and consistent across different regions after adjustment for mortality displacement. The impact of relative humidity appears to be different in regions, and may be a more important predictor of mortality in some areas.

## 1. Introduction

There is a growing literature on the impacts of exposure to heat on morbidity and mortality [1,2]. The physiological effects of heat on the thermoregulatory system are well documented and heat can, for example, cause dehydration, cardiovascular illness, endocrine diseases and kidney dysfunction, while respiratory effects are less well understood, but are still strongly associated with high temperatures [1]. However, the effects of weather on mortality in more northern regions are sparsely studied and therefore less is known about potential impacts of climate change, as well as contrasts to the more frequently studied regions, such as central and southern Europe and the US [1,2]. Several studies have been published on the differences in temperature susceptibility to heat and cold in warm and temperate climates [3–9]. However, so far, very few studies have assessed heat susceptibility on the arctic area borders [10,11]. Albeit, susceptibility to warm temperatures has been shown to increase over time in north Europe [12].

General estimates of mortality rates and temperature have shown a region-specific minimum mortality point that differs with climate [6,8,13]. The minimum mortality point has also been shown to change over time [7,14]. Some change is explained by socio-economic development, but it seem also be influenced by influenza and seasonality [6,11]. So far one study has proposed the effect of heat to depend on childhood early programming of climatologic conditions [15]. A few studies discuss the homogeneity in how different populations at widespread latitudes react to low temperatures and the heterogeneity in how they react to heat [9,13], and found that socio-economic conditions were associated with risks at both high and low temperatures [6,9]. It has been suggested that acclimatization to low temperatures takes place, but not to high temperatures [9]. Another study found heat thresholds to depend on climate [13].

The distribution of the temperature effects over days or weeks after exposure is a main issue, which is often accounted for by establishing distributed lag models [16]. In general, low temperatures act on longer lag times and high temperatures on shorter lag times, as well as the two major outcomes cardio-respiratory deaths often do [4,5]. It is even possible that a more resistant population has a longer delay between exposure and effect for warm temperatures [12]. The longer lag times also show on deficit in mortality rates if there is mortality displacement associated with the exposure effect [17,18]. The cumulative effects ranging over a number of lag strata, will therefore more accurately estimate the effect taking into account such tendencies if apparent [16]. Many studies of mortality associated with heat waves reveal greater consequences under the very extreme conditions where there is no relief during a longer period [11,19,20].

Studies on the impacts of ambient temperature often use either simply daily maxima, minima or mean temperatures, or a temperature index also incorporating indirectly levels of relative humidity or dew point temperature [2,4,5,13]. This can partly been motivated by the thermo-physiological impacts of reduced sweating capability with high relative humidity [2]. However, so forth the introduction of such weather indexes has shown to make a very small contribution, if any, to the model predictive performance [2,20]. Few studies have described the direct impacts of relative humidity on mortality rates, and the effect modification of high temperatures and high relative humidity.

We aim to study the similarities and differences in how the Swedish elderly population (aged 65 and above) responds to summer ambient temperatures in terms of population-level all-cause mortality in three regions of Sweden, ranging from the southern border of Scandinavia to the Stockholm region. Further, we aim to study the lag structure and the general effect of temperature generally, and assess the effect of high temperatures as well as the effect of low temperatures within summers. We also aim to study the effect of high and low ambient relative humidity, and the effect modification of high relative humidity and high temperatures.

## 2. Methods

Mortality data from the Cause of Death Register at the Swedish Board of Health and Welfare was collected for the period 1998–2005 as region specific daily mortality from natural causes (excl. external causes) in people aged 65 and above. The regions studied were Skåne (whole county), the Göteborg region (Göteborg and Mölndal municipalities) and Greater Stockholm. These regions represent large parts of Sweden’s densely populated areas from the south (Skåne) to the north (Greater Stockholm) corresponding to latitudes from 54° to 59° (Polar circle: 66.6°).

Temperature and relative humidity for the same time period were collected from the Swedish Meteorological and Hydrological Institute. In Skåne these observations were measured at the meteorological station at Jägersro, in Göteborg at Säve airport and in Stockholm at Bromma City airport.

All weather data were delivered as daily measurements. In the models we considered the daily mean values, since these have been found to best predict the mortality in previous time series studies [11]. We avoided unequal spacing of the observations by inputing missing values in the weather data as the mean of four surrounding observations. In the meteorological data for Göteborg the whole first summer (1998) was missing, and was therefore excluded from the analysis. Weather and mortality statistics and percentage of missing observations are presented in Table 1 and Table 2.

### 2.1. Statistical Analysis

In the first step of the analysis we established models including parametric smooth functions revealing the temperature-mortality relations for the three regions over the whole scale of temperatures (all-year). Counts of daily deaths in ages 65+ were assumed to follow an over-dispersed Poisson distribution in a generalized linear model in the R-statistical software (21). The lag strata of daily temperature were moving averages of lag 0–1, lag 2–6 and lag 7–13, while for relative humidity, lag 0 performed well as predictor according to the UBRE criterion. The UBRE criterion implemented in the mgcv package of R can be described as a generalized Akaike Information Criterion. Smooth functions (cubic splines) of temperature and relative humidity were controlled for by factors representing weekdays and national holidays, smooth functions for trends (between year changes) and seasonality (within year changes; unit days). The smooth function of seasonality was fit using periodic boundary conditions, and as all parametric smooth functions with fixed degree of freedom (df) [16,22]. The spline function of mean temperature was allowed 5 df in each lag stratum. Lag 0 of mean relative humidity was allowed 4 df, within year patterns (season) were allowed 5 df and between year patterns (trend) were allowed 4 df.

In the next step of the analysis we focused on the summer period, June-August, and patterns of mortality associated with daily mean temperature and daily mean relative humidity for the same lag strata and the same control for confounders as in the initial approach. Studying only the summer period we fitted general estimation equations with STATA [23] treating daily mortality between summers as independent observations and observations within summers as dependent on each other according to a covariance structure [24]. The covariance structure we chose to employ was a first order autocorrelation structure as has been used previously in such models [4,5,20]. In the models, the daily counts of mortality were assumed to follow a Poisson distribution. Temperature lag strata variables were introduced into the model as linear spline functions at 0th–50th, 50th–90th and 90th–100th percentile of the first lag strata variable (mean of temperature lag 0 and 1). The placement of the spline knots (50th and 90th percentile) were chosen to relax the often assumed linear relationship of mortality following high temperatures. Because the knots of the spline functions of the temperature lag variables were set at the same value the calculation of cumulative effects are more meaningful. The most intuitive and appealing interpretation is the net effect on a particular day induced by the past two weeks’ 1 degree mean increase of temperature above a knot (threshold).

The daily relative humidity was centered and modeled as a piecewise linear spline function with breakpoint at the mean value. Additionally, effect modification of high levels of relative humidity and high temperatures were modeled by a dummy variable that was non-zero when relative humidity levels were above the 80th percentile and the first lag strata variable of temperature (lag 0–1) were at levels above the 90th percentile. The estimates of relative humidity and temperature did not appeared sensitive to collinearity.

Moreover, we also controlled for several variables; trend as a parametric cubic spline function with 2 df per the eight years of the study, by a factor for month, a factor for weekday and by a indicator for national holidays.

The standard deviations and confidence limits that resulted from the models are robust to misspecification of the covariance structure within summers (the Huber/White/sandwich variance estimator was used instead of the generalized least squares estimator). Models were tested according to the Wald goodness of fit test based on the chi-square statistic and a residual diagnostics test was made to further evaluate the model fit. There were no obvious indications of model misspecification.

In addition, we assessed meta-estimates and homogeneity in effects using the Metareg package in STATA. All estimates for temperature are presented as relative risk (RR) per one unit increase in temperature with 95% confidence limits (CI).

## 3. Results

In Table 1 we present weather statistics for daily temperature and relative humidity for the whole study period, while Table 2 describes the range of daily temperatures and relative humidity in summer together with the daily mean and range of mortality (natural causes of deaths). The all-year mean temperature during the study period differs somewhat between regions, but the maximum daily temperatures are similar as well as the summer mean temperature. The standard deviation (std dev) of the daily mean summer temperature increases with increasing latitude as does the range of maximum and minimum temperatures. The summer mean relative humidity decreases a little with latitude. The range of the relative humidity in summer is similar in Stockholm and Göteborg, but much smaller in the most southerly situated region, Skåne. Generally, the variation of the relative humidity in summer seems to take on slightly greater values in the north and decrease the further south the region is situated.

**Figure 1.**
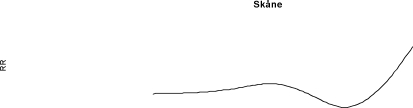
The exposure-response curve of temperature (°C) lag 0–1 and mortality (ages 65+) in the three regions studied. The x-axis corresponds to lag 0–1 temperature in the study region, and the y-axis to the relative effect on mortality. The vertical lines mark the temperature according to the scale on the x-axis.

In Figure 1 the smooth functions of the effect of temperature on mortality (lag 0–1) over the whole span of temperature are presented as risks relative to the minimal point of the curve for the three regions. The minimum mortality point of the curves is higher in the most southerly region and decrease with higher latitudes. As can be seen from the figure natural mortality in ages 65+ tends to increase more steeply for warm temperatures compared to cold temperature in lag strata 0–1. Note, however, that these curves are controlled for influences that act on longer lag times (the lag strata 2–6 and 7–13). The sharpest increase in relative risks is seen for to the most southerly situated region, while the others reach similar levels but over a larger range of temperature.

**Figure 2.**
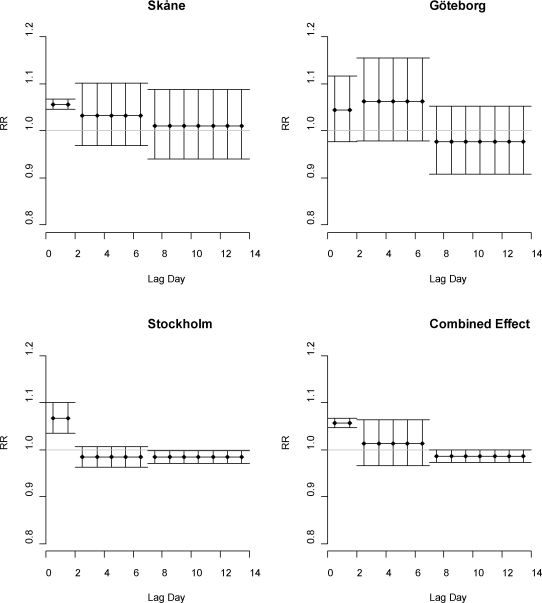
The distributed lagged relative risks of temperature per °C increase of lag strata temperature above 90th percentile (lag 0–1) with 95% confidence bounds as vertical lines. The x-axis corresponds to lag day, and the y-axis to the relative impact on mortality (65+ of ages). The horizontal line marks relative risk =1.

In Figure 2 we have plotted the distributed lagged relative risks of a unit increase in temperature lag 0–1, lag 2–6 and lag 7–13 in the study regions, together with the combined estimate for all regions.

Statistically significant relative risks can be seen in models for temperatures above the 90th percentile for Stockholm and Skåne (confidence interval not including 1). All the relative risks of lag 0–1 are of about the same sizes. On longer lag times the relative risks indicate increased mortality in Skåne and Göteborg, while lag 7–13 in Stockholm show statistically significant reduction in mortality rates the second week following exposure.

Below the 50th percentile of summer temperatures in Skåne there is a significant increase in mortality associated with decreasing temperatures in the lag strata, 7–13, of RR = 0.982 (95% CI = 0.969, 0.994). This effect is also indicated in the Göteborg region with about the same size of effect.

**Figure 3.**
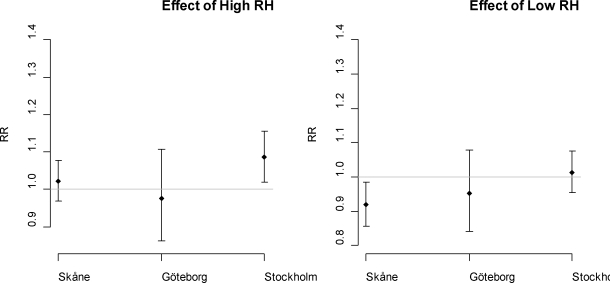

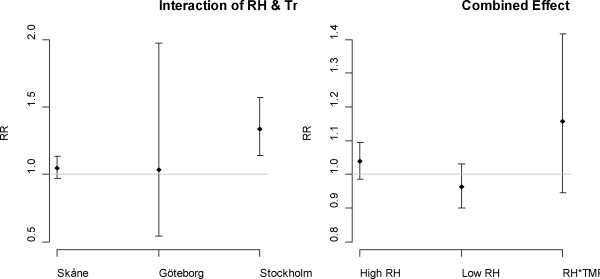
Above; the relative risks of daily relative humidity (lag 0) in categories high (above mean) and low per each 20 units increase of relative humidity in the three study regions. Below at left; effect modification of temperatures above 90th percentile and relative humidity above 80th percentile as indicator variable in the three study regions. Below right; the combined relative risk of relative humidity (for a 20 unit increase) and the combined relative risk of the effect modification with temperature (temperature above 90th percentile and relative humidity above 80th percentile). The vertical lines represent the 95% confidence width. The horizontal line marks relative risk =1.

The piecewise linear functions of relative humidity reveal no apparent increase in mortality associated with high effect of high level, with the exception of the Stockholm region where this effect is statistically significant. In Figure 3 the effects of high and low levels of relative humidity for lag 0, with high and low according to the mean level in each region, are shown with confidence limits for a 20 units increase. The effect below the threshold indicates increasing mortality with decreasing levels of relative humidity. The combined relative risks (*i.e*., for all three regions studied) for temperature are presented in Figure 2. The combined relative risk associated with 1 °C increase in lag 0–1 temperature is 1.057 (CI = 1.047, 1.068).

**Figure 4.**
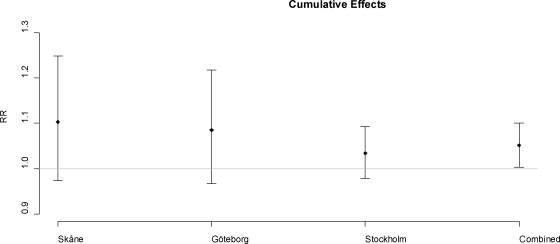
The cumulative relative risk per °C increase in two week mean temperature above 90th percentile in the three regions studied. The vertical lines represent the 95% confidence bounds. The horizontal line marks relative risk =1.

The estimated effect modification of temperatures above 90th percentile and humidity above the 80th percentile indicates increasing mortality rates in all regions after controlling for the main effects (TMP+RH +RH*TMP), but the only statistically significant effect modification was seen for the Stockholm region corresponding to a RR = 1.338 (95% CI = 1.141, 1.569), as can be seen in Figure 3. In Figure 3 the relative risk for relative humidity is also graphed as corresponding to a 20 unit increase in relative humidity for low (below summer mean) and high (above summer mean) levels of relative humidity. The cumulative relative risk of temperature over two week time is shown in Figure 4.

The RR corresponds to the effect on a single day per one degree increase in temperature over 2 weeks above the threshold of the 90th percentile. The combined effect for a one unit increase in temperature above the 90th percentile corresponds to a relative risk of 1.051 increase in mortality with confidence interval (CI = 1.003, 1.010).

## 4. Discussion

The relative risk of same or previous day temperature above the 90th percentile on mortality in age 65+ is rather similar in all three regions studied, even though the climate in the regions is somewhat different. We believe the populations in the three regions are quite homogeneous in terms of health and standard of living, since there are only small differences between regions in Sweden. However, the effect of high relative humidity and the effect modification of high level of relative humidity and high temperature were largest in the most densely populated area—Stockholm. Moreover, the susceptibility to cold temperatures during the summer appears greater in the south. The over-all combined relative risk estimate for the three regions and over two week show a rather significant increase in deaths rates in population age 65+ when temperatures increase above the 90th percentile, while the location specific cumulative effects do not. This is likely due to the weak signal in the lag times longer than lag 0–1.

Not surprisingly, the two regions with the most events per day, Greater Stockholm and Skåne, show more stable estimated effects; interpretations and contrasts in results between those models will therefore be more trustworthy.

The lag of the high temperature effect in Skåne and Göteborg are similar with indications of an effect at lag 0–6, while the positive effect for the Stockholm region is apparent only in lag 0–1. The latter is consistent with prior studies for Sweden [11,12]. The longer lag times in the two southerly situated regions together with indications of mortality displacement in the Stockholm region, may indicate that less susceptible people fall victim to higher temperatures in more southerly regions [12].

A prior study indicated no confounding with air pollution [12]. Because of this, possible effects of air pollution, in particular ozone, were not controlled for, but also because the local formation of ozone is small in Sweden and it is not strongly dependent on sunlight and temperature. The support for effects of other pollutants, such as particulate matter as confounders during summer is weak, with relatively low level in summer in Sweden. However, the lack of controlling for influenza in the all-year smooth functions may be a more serious concern for the all-year estimates and the cold impacts on mortality. However, we focused on shorter lag times, and the cold effects are generally seen on longer lag times [4,11,17]. Also, this study focuses on summer mortality and as is well known influenza is generally bound to the winter season in temperate countries.

The cumulative relative risks have big confidence limits with the exception of the combined cumulative effect. The cumulative measures of effects are unbiased even if there is colinearity between the explanatory lag strata variables of temperature [16], as well as they account for mortality displacement. Also, since the heat effect acts on shorter timescales, e.g., same or next day after exposure, the cumulative effects over two weeks are likely to have large variance due to greater uncertainty and smaller effects the following time that adds up to two weeks. The cumulative effect indicates that the effect of temperature is greater the further south the region is situated. This has also previously been found established in a larger European study [5].

These models do not take into account heat wave effects since the estimates do not necessarily correspond to a two week prolonged exposure, but rather to individual consecutive and non-consecutive lag strata exposure, and secondly because daily temperatures can be lower than the threshold, but still add up to fit the inclusion criteria in the model for moving averages. However, prior studies have shown that there might be an additional heat wave effect [11,19,20].

The less precise effect of weather on mortality in the Göteborg region may be because the predictors and definitions of strata fit less well here, possible because of climatologic differences due to more direct maritime influence from the Atlantic Ocean. However, to get combined effect estimates we needed to use the same predictors, lag strata and threshold, in all regions studied. It is of course possible that other weather predictors would have explained mortality even better in other regions, but the mean temperature, the lag strata and the threshold used have been shown to explain mortality in Stockholm well [11,12], and other generally small differences have been found between different predictors [2,20]. A more detailed approach taking many weather indexes into account might have revealed interesting differences, but still our approach allows the contribution of relative humidity to be different in different regions. In fact, our results highlight regional differences in the impacts of relative humidity on mortality rates, while some studies investigate heat related mortality in several cities or regions with meta estimates as an endpoint and utilizes a pre defined estimation procedure in all regions analyzed, with some but relatively little flexibility due to the wish to have comparability within the study. However, lag strata, biometeorological indices of temperature and relative humidity may to varying extents explain mortality rates at different locations with different climates, urban structures, demography, population characteristics, health conditions and behavior. Estimation of multiple exposure-response relationships assuming similar relationships (including lag structures) at different locations, e.g., for relative humidity and temperature and their interaction, give location insensitive estimates. Therefore, the use of biometerological indexes would be more or less appropriate in the regions studied depending on the impact and effect modification with relative humidity. The use of relative humidity as predictor adds valuable information on how it impacts mortality rates in the regions. However, temperature and relative humidity is correlated, and simultaneous introduction of these two predictors may induce bias as collinearity. In this study this did not appear to be a problem with the estimates appearing robust to changes in the models.

The overall results indicate similar heat susceptibility for temperature in different parts of Sweden. Interestingly cold related mortality in summers was observed in the most southerly of the regions, but not in the other regions. It is well known that southern Europe experiences more cold related mortality than northern Europe does [25]. If so, it might be due to mal-adaptation, in part influenced by behavioral factors that have been shown to be important to reduce mortality in the winter season [3].

The burden of temperature related mortality will likely increase with the ageing population in Sweden,; projected numbers from Statistics Sweden are a general 38% increase of ages 65–79 and a 57% increase in ages 80+ for Sweden by 2030. This would have large impacts in the annual attributed burden of heat according to the impacts estimates from this study. However, to understand how temperature related mortality will change with a changing climate we need to synthesize knowledge from a wide variety of locations, that when contrasted can be used to identify the unique contribution of climate change as a relative measure and the contribution of climate change as an absolute measure (not depending on the change but the actual level). More research on both cold related and heat related mortality as well as climatologic determinants of death and disease patterns such as seasonality is needed to prevent increases in mortality in the future as a consequence of climate change.

## Figures and Tables

**Table 1.** Descriptive statistics for daily weather during the study period in the three regions with temperature measured in °C and relative humidity in %.

**Variable**	**Mean daily temperature**	**Daily temperature**		**Daily relative humidity**		

		**Minimum**	**Maximum**	**Mean**	**Minimum**	**Maximum**

**Skåne**	9.0	−11.4	24.4	81.0	39.1	99.0
**Göteborg**	8.9	−15.1	24.9	76.8	31.0	99.0
**Stockholm**	7.3	−21.7	25.2	75.3	33.1	97.3

**Table 2.** Descriptive statistics for daily summer temperature and relative humidity and daily mortality in the population aged 65 and above, over the study in the three regions studied.

**Variable**	**No obs**	**Mean**	**Std dev**	**Minimum**	**Maximum**	**Missing**

**Skåne**						
**Temperature**	736	16.7	2.6	9.7	24.4	<1%
**Rel. Humidity**	736	76.0	8.0	52.0	96.4	<1%
**Number of deaths**	736	22	5.1	9	38	

**Göteborg**						
**Temperature**	644	17.0	2.8	9.4	24.9	<1%
**Rel. Humidity**	644	71.8	8.8	38.1	93.4	<4%
**Number of deaths**	644	10	3.4	1	21	

**Stockholm**						
**Temperature**	736	16.8	2.9	8.7	25.2	
**Rel. Humidity**	736	69.2	11.2	34.9	93.9	<6%
**Number of deaths**	736	22	5.3	7	39	<4%

**Table 3.** Percentiles (50th and 90th) of the summer daily mean temperatures of lag 0 and 1, as well as the 80th percentile of daily mean relative humidity for three study regions.

**Temperature lag 0–1:**	**50th percentile**	**90th percentile**	**80th percentile of relative humidity**

**Skåne**	16.4 °C	20.1 °C	82.8
**Göteborg**	16.6 °C	21.0 °C	79.1
**Stockholm**	16.4 °C	20.7 °C	79.1
